# Microbial Succession on Microplastics in Wastewater Treatment Plants: Exploring the Complexities of Microplastic-Microbiome Interactions

**DOI:** 10.1007/s00248-024-02422-y

**Published:** 2024-08-12

**Authors:** Klaudia Kwiatkowska, Paulina Ormaniec

**Affiliations:** Department of Environmental Technologies, Faculty of Environmental Engineering and Energy, Cracow University of Technology, Warszawska 24, 31-155 Kraków, Poland

**Keywords:** Microplastic, Biofilm, Microorganisms, Wastewater treatment plant, Sewage sludge

## Abstract

Despite some effectiveness of wastewater treatment processes, microplastics accumulate in sewage sludge and their further use may contribute to the release of plastic microplastics into the environment. There is an urgent need to reduce the amount of microplastics in sewage sludge. Plastic particles serve as solid substrates for various microorganisms, promoting the formation of microbial biofilms with different metabolic activities. The biofilm environment associated with microplastics will determine the efficiency of treatment processes, especially biological methods, and the mechanisms of organic compound conversion. A significant source of microplastics is the land application of sewage sludge from wastewater treatment plants. The detrimental impact of microplastics affects soil enzymatic activity, soil microorganisms, flora, fauna, and plant production. This review article summarizes the development of research related to microplastics and discusses the issue of microplastic introduction from sewage sludge. Given that microplastics can contain complex composite polymers and form a plastisphere, further research is needed to understand their potential environmental impact, pathogenicity, and the characteristics of biofilms in wastewater treatment systems. The article also discusses the physicochemical properties of microplastics in wastewater treatment plants and their role in biofilm formation. Then, the article explained the impact of these properties on the possibility of the formation of biofilms on their surface due to the peculiar structure of microorganisms and also characterized what factors enable the formation of specific plastisphere in wastewater treatment plants. It highlights the urgent need to understand the basic information about microplastics to assess environmental toxicity more rationally, enabling better pollution control and the development of regulatory standards to manage microplastics entering the environment.

## Introduction

Socio-economic progress on a global scale constantly introduces new substances into the environment, which appear in our surroundings almost immediately. Chemicals are present in every aspect of the environment, and in the materials, we come into contact with every day, such as home, workplace, and food. Nowadays, we increasingly come across information about emerging pollutants (EPs). Although the term EPs is relatively new, their presence in various elements of the environment is not a new phenomenon. The first reports of EPs can be attributed to Rachel Carson, who in 1962 showed that compounds commonly used to control pests contribute to the death and extinction of many birds [60]. In recent years, special attention has been paid to microplastics (MPs) due to their toxic impact on the environment.

The definition of MPs is diverse and depends on its sources. However, it is assumed that MPs are plastic particles that are insoluble in water. According to popular belief, MPs are plastics that do not exceed 5 mm in size (Frias and Nash, 2019). MPs are often divided into two categories based on their size: *large microplastics*, which range in size from 5 to 1 mm, and *microplastics*, which are particles smaller than 1 mm to 1 µm (ISO 24187:2023).

Depending on their origin, MPs are divided into secondary and primary. Primary MPs are produced in microscopic size and intentionally added to other products or production processes. They have been found in body care cosmetics, as confirmed by Dąbrowska et al. [10]. Many primary MP particles are often released directly into the environment, for example, during the sandblasting process. Modern sandblasting methods increasingly use MPs instead of traditional sand. As a result, these MP pieces can be carried by the wind and then settle in water reservoirs [1]. Another form of primary MPs is industrial raw materials. Small, colorful plastic pellets are used in industries around the world and are used to produce larger plastic components [38]. Although primary MPs are present in the environment and there are many sources, they are not as common as secondary MPs. Secondary MPs are produced by the breakdown of larger plastic particles. This degradation may be caused by mechanical, chemical, or biological factors. The degradation process of polymers involves changing their characteristics or chemical structure, which may lead to fragmentation. Fragmentation of polymers occurs by weakening their structure and results in the formation of smaller particles [75]. For example, tire abrasion is a large source of secondary MPs entering the aquatic environment [67]. Washing fabrics made of synthetic materials such as polyester, nylon, or acrylic can lead to the release of MPs into the environment. This occurs when synthetic fibers abrade and release MPs into the water during the washing process, particularly when detergents and intensive spin cycles are used [11].

One of the first reports on the occurrence of MPs in the natural environment dates back to 1972. Carpenten and Smith [4] reported the presence of small plastic particles in the western Sargasso Sea at an average concentration of 3500 pieces (290 g) per km^2^ of surface. Currently, there is a lot of data on the extent of MP pollution in aquatic, terrestrial, and air environments. However, due to the lack of unification of the method of MP characterization, these studies are difficult to compare. Nevertheless, MPs have been confirmed to occur in virtually all aquatic ecosystems worldwide. They have been reported in freshwater environments such as rivers, lakes, and water ponds [6, 16, 41]. Therefore, their occurrence has been confirmed in seas and oceans [25, 31]. Furthermore, the presence of MPs has been confirmed in aquatic sediments and coastal sands [29],Urban-Maliga et al. 2020).

MPs can enter soils through various means, including agricultural activities, the use of fertilizers and pesticides, and environmental pollution such as illegal garbage dumps [18]. Additionally, research has shown that MPs are present in the atmosphere, both in urban and remote rural areas. Airborne MPs can be transported over long distances by wind and precipitation [20]. MPs have been detected even in remote areas of the Arctic. Despite the Arctic being one of the most isolated and inaccessible areas on Earth, research has shown the presence of MPs in the ice, sea waters, and sediments in the area [3, 48].

Extensive studies of the natural environment, including marine and fresh waters, soil, and atmosphere have revealed the presence of MPs in various biological environments. MPs have also been detected in human urine samples and kidney tissues [51]. Moreover, studies have shown the presence of MPs in breast milk samples, suggesting the possibility of these particles penetrating physiological barriers and accumulating in tissues and body fluids [55]. These observations highlight the importance of understanding the mechanisms of entry and distribution of MPs within the human body and the potential health effects associated with exposure to these substances.

MPs have a significant environmental impact due to their ability to adsorb various contaminants, including hydrophobic organic pollutants (HOCs), heavy metals, pharmaceuticals and antibiotics, and personal care products [30],Amelia et al., 2021, [74]. These interactions occur through a range of mechanisms, including hydrophobic interactions, electrostatic interactions, hydrogen bonds, and Van der Waals forces. The characteristics of MPs, including polymer type, UV-induced surface modifications, and the presence of oxygen-containing functional groups, significantly affect these interactions. MPs increase the exposure of organisms to HOCs, leading to bioaccumulation and biomagnification in the food chain, which poses ecological and health risks [53]. Additionally, the shape and size of MPs influence the development of biofilms, which can alter the dynamics of contaminants and microbial communities in aquatic environments [59]. It is therefore crucial to understand these interactions in order to assess the actual impact of MPs on ecosystems and human health, as well as to develop effective methods for the removal of MPs. It is well known that sewage sludge has major agricultural applications and carries microorganisms directly into the soil. It is known that MPs can act as carriers of various micropollutants, including microorganisms.

Microorganisms, as with any type of surface, attach to MP surfaces and colonize them as soon as material enters, forming MP biofilms called the plastisphere. The plastisphere can be considered a new microecosystem, as microbial communities consist of a variety of bacteria, fungi, viruses, archaeons, algae, and protozoa. Many factors influence the composition and development of the biofilm on MPs, including environmental conditions, substrate type, particle size, and surface properties. Therefore, plastisphere microorganisms are also phenotypically diverse, representing a wide range of preferred environmental conditions. Interspecies interactions, such as competition and levels of gene transfer, affect the structure, stability, and behavior of biofilms and can affect plastisphere colonization and biodegradation [7]. When MP particles enter the wastewater treatment plant (WWTP), they can interact with bacteria actively involved in treatment and negatively affect the efficiency of the processes themselves. Micro- and nanoplastics inhibit the activity of activated sludge, affect the efficiency of methane production, and reduce the diversity of biological communities and the abundance of key groups of microorganisms. Studies suggest that MPs affect the efficiency of inorganic nitrogen conversion, leading to ammonia accumulation [56]. Thus, plastic particles alter the processes mediated by microorganisms, especially the cycling of nitrogen compounds. In addition, polyethylene nanoplastics with a positive surface charge have a high affinity for activated sludge (which has a negative charge), so microbial cells can be damaged, free radicals can be formed, and sludge bioflocculation can occur. Current methods of identifying MPs still do not provide a complete answer to the type of impact of plastic particles on the effectiveness of wastewater treatment technologies. This calls into question the ability of European institutions to meet the increasingly high environmental demands placed on the water and wastewater industry [57].

## Microplastics in Wastewater Treatment Plant

In the context of WWTPs, MPs are becoming an increasingly important research topic because the treatment process can have a significant impact on their presence and fate in the natural environment. There is therefore a need for an in-depth analysis of the role of biological processes in the context of MPs in WWTPs, which may contribute to a better understanding of this problem and the development of more effective pollution management strategies.

Analyzing research on the content of MPs in WWTPs, significant disproportions in the concentrations of these pollutants are visible. Differences in MP concentration levels may be related to the geographical location of the treatment plant, which significantly affects the characteristics of the wastewater streams flowing into it. WWTPs located in areas with varying degrees of urbanization tend to be exposed to various types of pollutants, which directly determine the content of MPs in the treated wastewater. Wastewater coming mainly from residential areas may carry different types of MPs compared to those coming from industrial or agricultural areas. Additionally, variations in the treatment technologies used and their effectiveness may significantly affect the abundance and characteristics of MPs present in treated wastewater streams. Consequently, the variation in the content of MPs in WWTPs may be the result of the interaction of many factors, including the location of the treatment plant, the specific nature of the incoming wastewater, and the treatment process technologies used.

Determining the load of MPs in sludge is important because of their persistence and presence in solid waste, which is often distributed over land areas and can affect a variety of natural ecosystem properties and processes. For example, the presence of MPs has been shown to affect the biological process of anaerobic digestion in sediments. A study of MPs, specifically polyethylene, by Wei and other researchers found that the presence of 100 or 200 MPs/g of activated sludge significantly reduced the production of methane, a key product in the anaerobic digestion process [70]. This work focuses on presenting the MP content of final sewage sludge, which is often later used in agriculture. The concentration of MPs in sewage sludge is studied all over the world. MPs commonly found in WWTPs are removed satisfactorily from wastewater,however, most of them end up in sewage sludge [66]. Table 1 shows the amounts of MPs in sewage sludge from WWTPs in different countries. A review of the content of MPs in sewage sludge aims to highlight the quantity and occurrence of these particles, which results in the need to understand their potential role as transporters of microbial organisms.

**Table 1:** Microplastic content in sewage sludge in selected wastewater treatment plants worldwide

Location	Microplastics concentration [× 10^3^ pcs MP/kg ds]	Limit size [µm]	Sources
Spain, Europe	133.0 ± 59.0	36–4720	[14]
Norway, Europe	6.07	50	[42]
Finland, Europe	186.7 ± 26.0	20	[66]
Germany, Europe	1–24	< 500	[46]
Iran, Asia	129 ± 17	37	[50]
China, Asia	7.70 ± 0.8–46.0 ± 10.7	37	[39]
Morocco, Africa	36.0 ± 9.7	500	[15]
Mauritius, Africa	14.75 ± 8.61	250	[54]
Canada, North America	228–1353	80	[63]
Chile, South America	18–41	8	[9]

In one of the Spanish WWTP, the occurrence of MPs was investigated in primary and secondary effluent and mixed sludge, as well as in thermally treated dried sludge sold as a soil additive. It was found that the final sludge contained 133 ± 59 particles/g dried solids (ds), which was not significantly different from the value obtained for thermally dried sludge used as a soil additive [14]. Research carried out at eight different WWTPs in Norway showed that the average content of MPs in sewage sludge was 6077 MP/kg ds. The most commonly identified form of plastic was beads, followed by fragments, fibers, and glitter. Plastic sizes ranged from 54 (detection limit 50 µm) to 4987 µm, with an average size of 644 µm [42]. Telavitie et al. conducted a detailed study of MP removal in a tertiary WWTP in Finland. The research focuses on evaluating the efficiency of MP removal processes at the different treatment stages. The results show a significant reduction of MPs in the wastewater treatment process, but confirm the fact that sludge accumulates MP particles. The study showed 186.7 ± 26.0 MP/g ds (Telavitie et al. 2017). In their study, Mintening et al. reported MPs in sewage sludge ranging from 1000 to 24,000 MPs/kg ds. They also noted that none of the sewage sludge samples tested contained MPs > 500 mm; it was MPs < 500 mm that were detected in all sewage sludge samples [46].

Petroody et al. conducted a study on the presence of MPs in the sludge produced by the Sari WWTP in northern Iran. Dry sludge from the primary clarifier, after thickening, after digestion, and after dewatering was tested. The dewatered sludge contained 129 ± 17 MP particles larger than 37 µm per g dry weight. Of all the MPs identified, up to 87.5% were fibers, the majority of which were polyester fibers. Among the remaining MP particles, polyethylene particles predominated. A comparison of tests on different types of sludge showed that the concentration of MPs in dewatered sludge was 54% lower than in the digestion product. It is possible that the removed MPs are returned to the treatment system with the leachate from sludge dewatering [50]. Studies from 28 different WWTPs in China showed the average content of MPs in each of them. Analysis of all WWTPs showed that the lowest average MP content in dewatered sludge was 7.70 ± 0.8 MP/g ds, while the highest was 46.0 ± 10.7 MP/g ds. Thus, it was found that MPs in sludge from WWTPs in China exceed those in freshwater sediments by one to two orders of magnitude. The average concentration of MP particles in the samples was 22.7 ± 12.1 MP/g dry sediment, which allows us to estimate that approximately 156 trillion MP particles from Chinese sediments enter the environment annually [39].

Studies on sludge from Moroccan WWTP showed an average of 40.5 ± 11.9 × 10^3^ MPs/kg ds and 36 ± 9.7 × 10^3^ MPs/kg ds in fresh and dewatered sludge, respectively. Due to the dewatering process of sludge in drying beds, an MP loss of less than 500 µm was assumed. Using the pyrolysis–gas chromatography/mass spectrometry technique, polymers such as polystyrene, polypropylene, polyamide, and polyethylene were identified. The aim of the study by Ragoobur et al. was to investigate the presence of MPs in agricultural soils, sewage effluent, and sewage sludge in Mauritius. The mean concentration of MPs in sewage sludge was 14,750 ± 8612.9 MP/kg. They classified the isolated MPs by size into ranges: 5–3 mm, 3–1 mm, 1–0.5 mm, and 0.5–0.25 mm, with 90% of the MPs being smaller than 0.5 mm [54].

In their study, Sivarajah et al. reported on the occurrence of MPs in 22 Canadian WWTPs. The concentration of MPs ranged from 228 to 1353 particles per gram of dry solids. The median for all samples was 636 particles per gram of dry solids. Fibers were the most commonly identified MP shape in all samples, with frequencies ranging from 73 to 92%. Particles with a minimum size of 80 µm were tested [63].

The aim of the research carried out in Chile was to assess MP contamination of soils through the use of sewage sludge for agricultural purposes. The MP content of the sewage sludge ranged from 18 to 41 particles per gram of dry solids (median = 34 MP/g ds). Most of the MPs observed were synthetic fibers. It was observed that MPs accumulate in the soil over time and increase with subsequent sludge applications [9].

Accumulated MPs in sewage sludge vary considerably in size, shape, or the type of polymer from which they are made. Depending on these characteristics, MPs can adsorb other contaminants on their surface. However, the most common particles found in sewage sludge are those from consumer products, such as textile fibers or plastic packaging fragments [19]. Depending on the stage of wastewater treatment, there are differences in the removal of MPs, both due to their size and the type of plastic. Primary treatment shows a higher removal efficiency of MPs, especially fibers, due to flocculation and sedimentation processes. Plastic fragments are most effectively removed by secondary treatment, while plastic pellets are most effectively removed by tertiary treatment. Tertiary processes are particularly effective in removing very small MP particles, suggesting that their use is necessary for the complete removal of MP contamination [40]. In addition, the efficiency of removal of MPs from wastewater, and thus their accumulation in sludge, is highly dependent on their size. Primary and secondary treatment processes are more effective in removing MPs of different sizes than tertiary processes [58]. Large-size, low-density MPs and films are easily removed by flotation and fat-removal processes. In contrast, high-density pellets tend to sink to the bottom [40].

The data presented from various WWTPs located around the world show how much of an environmental risk the use of sludge for agricultural purposes is. Along with sewage sludge, MP directly enters the environment. The MPs found in sewage sludge are characterized by a wide range of shapes, sizes, and the types of polymers from which they are made. It is likely that, depending on the different morphological characteristics of MPs, the interactions between them and microbiology will vary. Unfortunately, to date, there is a lack of data that clearly shows the environmental impact of these pollutants. There are reports where MPs are treated as a potential source of microorganism transfer on their surface. The following section presents the literature reports to date focusing on microplastic-microorganism interactions.

## Biological Impacts of Microplastics

MPs are a ubiquitous water pollutant around the world. For this reason, they have been the subject of intense research in recent years. Of particular interest are the interactions between MPs and microorganisms. The discovery of a biome specific to plastics, the so-called plastisphere, makes it possible to observe the interactions taking place in this peculiar biocenosis and thus periodize potential sources of microbial contamination [65]. MPs are not only contaminants in aquatic habitats around the world, but also occur in wastewater. Therefore, WWTPs are point sources of MP occurrence [33]. MPs provide a stable habitat for the growth of various species of sewage bacteria, including pathogenic and antibiotic-resistant species [35]. In addition, MPs interact with ubiquitous biofilms. MPs attach at the water-biofilm interface or penetrate the biofilm matrix. Thus, they can accumulate or adsorb in biofilms, where they undergo transformation processes. The succession of microorganisms in the plastisphere and their potential to degrade plastics is still unclear. Microorganisms can colonize substrate surfaces by effectively adhering to the surface within a few hours, followed by microbial growth and maturation. Meanwhile, microbial colonization and biofilm succession on plastics can affect their physicochemical properties [45].

### Biofilm

Many bacterial taxa are capable of biofilm formation. A biofilm, which is a specific biological membrane composed of a diverse group of microorganisms, can be controlled by multiple genetic pathways. As a result, microorganisms achieve increased resistance to stressors. Biofilm formation is caused by microbial cells of a single or heterogeneous species. Biofilm protects microorganisms from stressful environmental conditions, toxic effects of chemicals, and antimicrobial substances [5].

A biofilm is a specific ecosystem, consisting of a colony of bacteria in an exopolysaccharide matrix that has the ability to attach to foreign surfaces. It is a community of microorganisms, capable of living and reproducing as a colony unit. The ability to form a biofilm structure serves both to protect against environmental factors and to allow colony expansion. The composition of the biofilm includes 10% microbial mass and 90% water [27]. Matrix-forming polysaccharides account for 50–90% of the total organic component of biofilms. Polysaccharide chains are intertwined with each other in a dense, reticulate structure. The hydroxyl groups of the polysaccharides increase mechanical strength by interacting with each other [62]. The biofilm architecture can contain positively charged ions, such as Ca^2+^ or Mg^2+^, which allow biofilm growth up to 300 µm thick. Biofilms can also contain uronic acids such as d-glucuronic, d-galacturonic, and mannuronic acids, which give them anionic properties. The anionic properties allow the binding of divalent cations and provide greater binding strength for mature biofilms [13].

The different charges and ions in the biofilm ensure the structural integrity of the EPS (extracellular polymeric substance), which gives biofilms the ability to withstand environments with extreme abrasive forces. Bacteria growing in the biofilm are sedentary and are responsible for most of the physiological processes occurring in the biofilm environment. Sedentary bacterial biofilm communities are characterized by different growth rates, gene expression, transcription, and translation. The formation of a three-dimensional biofilm architecture is a multi-step process and includes adsorption, adhesion, microcolony formation, maturation, and dispersion. The characteristics of the substrate can have a significant impact on the speed and degree of attachment of microorganisms. Studies confirm that biofilms form faster in rougher and more hydrophobic materials [37].

The situation becomes more complicated when one considers that any substrate placed in a liquid environment acquires a coating consisting mainly of proteinaceous material present in the liquid environment. In addition to the properties of the substrate, the properties of the cell surface are also important. For example, the presence of strands, fimbriae, or glycocalyx can affect the rate of attachment of microorganisms. This is because the microbial cell must overcome the repulsive forces common to all materials, and the protrusions allow the cell to remain attached until more permanent attachment mechanisms emerge.

It has also been shown that the hydrophobicity of the cell surface is very important for adhesion. In the initial phase of biofilm formation, microorganisms are loosely and reversibly attached to the surface. The microorganisms then change orientation, lie flat on surfaces, and attach themselves irreversibly to the surface. Bis-(3′-5′)-cyclic dimeric guanosine monophosphate (c-di-GMP) is an intracellular signaling molecule that plays an important role in the early stages of biofilm formation by reducing flagella-mediated swimming motility and increasing biofilm matrix production [68]. The early stages of biofilm formation involve the conversion of bacterial cells with low concentrations of c-di-GMP that were not initially in contact with the surface [73]. Shortly after the successful adhesion of microorganisms to the surface, the microorganisms begin to multiply and aggregate within the self-produced EPS, leading to the formation of microcolonies in the presence of high concentrations of c-di-GMP. EPS plays a key role in biofilm maturation as it helps microorganisms attach to surfaces, stabilize the three-dimensional structure of the biofilm, cluster cells, and protect against various stressors.

**Fig. 1:**
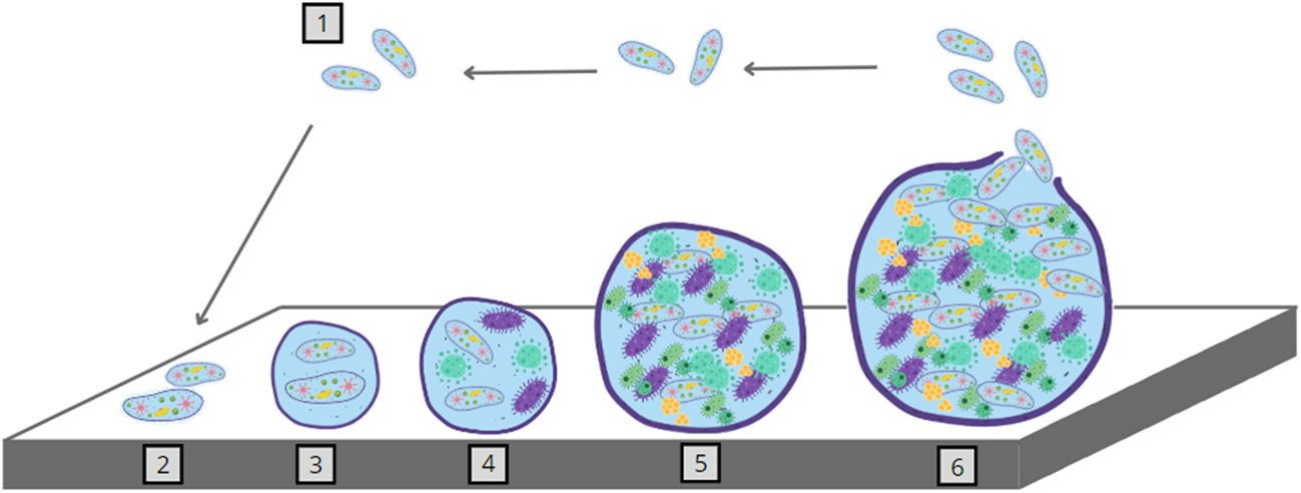
Stages of biofilm formation: (1) free-living bacteria, (2) reversible adhesion, (3) irreversible adhesion, (4) formation of microcolonies, (5) mature biofilm form, (6) dispersion and migration of cells from the biofilm

A mature biofilm can acquire a three-layered structure in which microorganisms are arranged according to their air tolerance and metabolic rate. The layering includes an inner regulating layer, a middle layer that constitutes the basis for the development of microorganisms, and an outer layer formed by forms of microorganisms ready to leave the biofilm. EPS forms a scaffold that holds the biofilm together and thus aids in cell-to-cell communication and provides the adhesion and cohesion forces necessary for biofilm formation. In addition, EPS helps nutrient cycling by maintaining the availability of deoxyribonucleic acid for horizontal gene transfer and acts as a protective barrier against oxidative biocides, antibiotics, and ultraviolet radiation [57]. Figure 1 shows a diagram of biofilm formation. Bacteria are commonly detected communities on the surface of MPs. Proteobacteria, especially Alphaproteobacteria, Gammaproteobacteria, and Betaproteobacteria are the dominant bacterial communities in the plastisphere [68].

### Microplastics vs. Microorganisms

Microorganisms are known to colonize MP surfaces. Interactions between microorganisms and MPs depend on MP surface properties (including size, shape, surface roughness, and hydrophobicity), as well as environmental factors such as temperature, pH, and ionic strength.

In the case of bacteria, the initial interaction occurs through electrostatic forces and depends on MPs size, chemical composition, and surface modification. MPs can provide a suitable substrate growth area for the microbial community and important nutrients for their growth (which are adsorbed on their surface from the environment, such as metal ions such as zinc, iron, and copper). Bacterial growth is facilitated by the rough surface of weathered MPs, during which biofilms form [28]. Many microorganisms have been found on the surface of MPs, including bacteria such as *Aeromonas*, *Rhodococcus*, *Pseudomonas*, *Enterobacter*, *Halomonas*, *Mycobacterium*, *Photobacterium*, and *Shigella,* as well as fungi. It is noteworthy that bacterial communities on MP (“plastisphere”) vary significantly depending on the type of aquatic environment and also vary depending on the composition and properties of MP (e.g., polyethylene vs. polypropylene, biodegradable vs. degradable), indicating selectivity of colonization.

Micro- and nanoplastics can be used as carriers for pathogens to migrate over long distances. In most cases, there is electrostatic repulsion between pathogens and MPs, since both have negatively charged surfaces [17]. However, the repulsion is overcome by the complexity of the pathogen’s strands, proteins, and hydrophobicity of the cell surface. The characteristics of the pathogen also affect its co-transport mechanisms with MPs. Large specific surface area and small particle size are key factors for MP adsorption and transport. The increase in bacterial adsorption on MPs with decreasing particle size can be explained by a larger specific surface area. However, the hydrophobicity and crystallinity of MPs’ surfaces have also been shown to reduce biofilm adhesion. Temperature affects the interaction of MPs with pathogens mainly by changing the physicochemical properties, physiological properties, and thermodynamics of adsorption [72].

Many researchers have found that temperature affects pathogen adhesion to solid surfaces and migration behavior in the subsurface environment. There are studies that illustrate the temporal and successional dynamics of biofilms and clearly show that increasing temperature plays a role in the formation of plastic-specific microbial communities. Environmental temperature affects bacterial surface charge, hydrophobicity, and outer membrane components (e.g., lipopolysaccharides and flagella), which explains the differences in pathogen adhesion to MP surfaces. The influence of environmental factors on the interaction and synergistic transport between MPs and pathogens is extremely complex. Atmospheric processes, such as photo-oxidation and UV radiation, affect interactions as a result of changes in the morphological structure and surface characteristics of MPs. Ionic strength, pH, and temperature change the surface charge of MPs and pathogens, thus affecting their adsorption and transport capacity [12].

MPs provide a stable and protective habitat for various wastewater bacteria, including pathogenic and antibiotic-resistant species. Therefore, they have the potential to carry these bacteria through wastewater treatment steps into the environment and over long distances. MP-associated biofilms have been proven to be an important source of pathogens and antibiotic resistance genes in natural waters. Municipal WWTPs are one of the main recipients of MPs from our daily operations. Although wastewater treatment plants act as a barrier to MPs entering the aquatic environment by removing MPs from wastewater (up to 99%), studies show that no wastewater treatment technique ensures complete retention of MPs, so WWTPs are seen as a pathway for MPs to enter the aquatic environment (Sharma 2023). Most municipal WWTPs include biological treatment based on the activated sludge process. Wastewater entering WWTPs contains a wide range of human microflora, including pathogenic bacteria. Recent studies of biofilms have shown an increase in bacterial species richness and taxon abundance during wastewater treatment processes, suggesting that WWTPs may play a significant role in modifying the plastisphere. In addition, MPs may act not only as transporters, but also as diffusion points for antibiotic resistance among bacteria, including phylogenetically distinct species [34].

WWTPs are a peculiar ecosystem with different groups of microorganisms. Many of them are species responsible for human diseases. Therefore, close and prolonged contact with environmental bacteria can increase the spread of antibiotic resistance through interspecies horizontal gene transfer (Kruglova, 2022). Studies show that MP surface influences bacterial colonization by selecting specific groups. This relationship has a major impact on the bacterial community. Therefore, the prevailing environmental conditions during wastewater treatment processes seem to be less important. However, if MP particles enter the WWTPs, they can interact with bacteria actively involved in treatment and thus negatively affect the efficiency of the wastewater treatment process (Grace, 2023). Studies show that no single wastewater treatment technique is sufficient to remove MPs present in wastewater. For this reason, WWTPs are seen as one source of MPs entering the environment [8]. WWTPs use a biological treatment process, which is based on the activated sludge process. Therefore, the presence of MPs at this stage can affect its efficiency (Lares et al. 2018). It is worth considering that colonization and distribution of microplastic-associated microorganisms in wastewater biofilms depend on interactions between exopolysaccharides segregated by bacteria and factors that are not yet known [26]. The biological process remains the most attractive approach in WWTPs because microorganisms have a remarkable ability to absorb organic compounds, thereby reducing wastewater contamination. At the same time, penetrating MPs can be considered vectors or carriers of toxic substances, including persistent pharmaceutical pollutants (POPs) and heavy metals. This is due to the large specific surface area of MPs resulting from their small dimensions and irregular shapes, as well as their lipophilic nature. When the properties, parameters, and behavior of wastewater change, they can form an impermeable biofilm that protects microorganisms from destruction. Several studies have shown that the formation of biofilms on the surface of MPs will increase survival because they are much more resistant than those suspended in the treatment system. Additionally, they are more resistant to sudden changes in environmental variables such as pH concentration and temperature [30, 66, 72].

Therefore, the study of microbial community development and metabolic functions in biofilm is extremely important. The MP surface itself ensures the survival and growth of bacteria, creating a specific ecosystem. Studies have shown that the microbial community varies depending on the stage of wastewater treatment, including primary, secondary, and tertiary treatment. During mechanical cleaning, microbial colonization forms a biofilm with microorganisms resistant to adverse environmental conditions. Some microorganisms prefer to attach to the MP surface as pioneers. This transition leads to changes in community adaptability, resulting in increased resistance to disinfection processes during wastewater treatment. This raises concerns about the use of MPs as potential carriers of microbial contaminants entering the environment [23].

## Sewage Sludge as a Potential Transporter of Microplastic Biofilm into the Soil

**Fig. 2:**
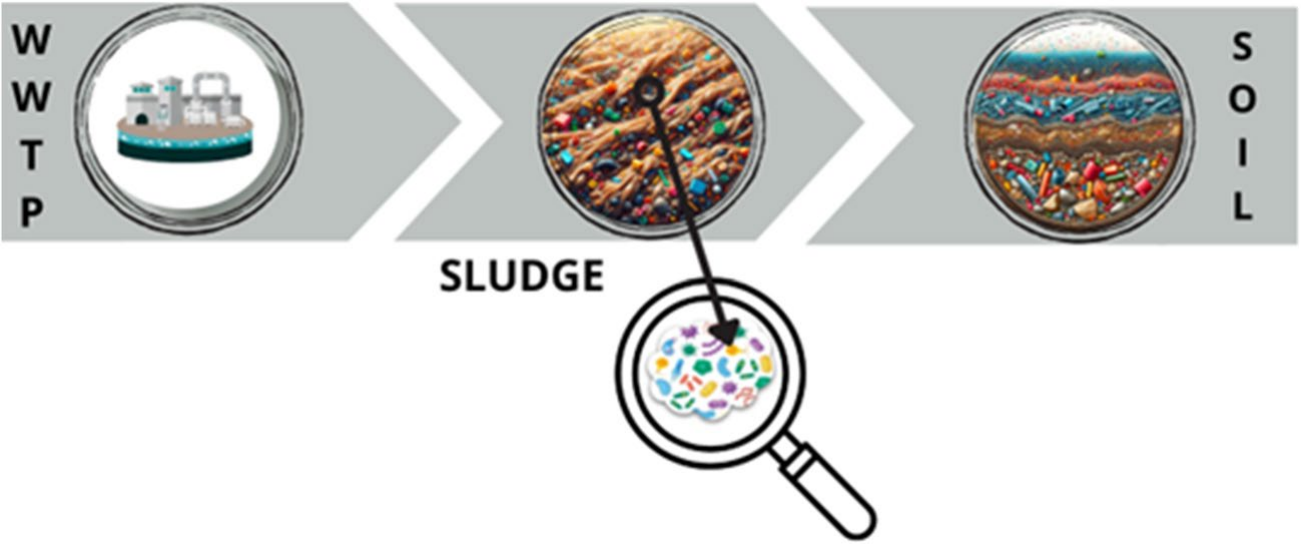
Distribution of biofilm-associated microplastics into the soil environment

Sewage sludge is a biological residue. For this reason, they are often used in agriculture as an alternative disposal technology for waste generated after wastewater treatment [64]. Therefore, sewage sludge can be a source of direct MP input into the soil as shown in Fig. 2. Sewage sludge is a by-product of WWTPs, which are widely used in agriculture. There is significant concern about the appearance of contaminants, including MPs, which can pose a serious danger to the environment [36].

Composted sewage sludge (CSS) has gained attention as a potential agricultural fertilizer. Its use increases microbial activity in the soil but can also lead to increased chemical and microbial risks [39]. Sludge is a habitat where the potential presence of human pathogens entering from WWTPs can contaminate products grown in the soil. Indirectly posing a risk to consumers. In addition to the presence of pathogenic microorganisms, sewage sludge can contain other organic and inorganic contaminants, as well as a biofilm associated with MPs [43].

Due to the widespread use of sewage sludge in the agricultural sector, MPs are often present in fertilized soil. This causes changes in both the nutrient content of the soil and affects soil microorganisms [2]. Studies show that the introduction of plastic microbeads into the soil can not only change soil physicochemical properties and microbial populations, but also affect soil enzymatic activity (van den [69]). Microorganisms, which decompose organic matter in the soil, significantly interact with MP pollutants. The complex microbial network of bacteria, fungi, protozoa, and algae plays a key role in agroecosystems. Imbalances and changes in the functioning and structure of microbial communities can have consequences for the entire system, and thus for crops in agricultural fields. In addition, plants are linked to the soil microbiome and fauna. They depend on this network for various functions, such as growth, and development, protection from pathogens, productivity and yield, and nutrient mobilization (Shafer et al. 2022).

Understanding the morphology of MPs in sewage sludge is essential for assessing the impact of sludge treatment processes on MP morphology. However, the morphology of MPs in sewage sludge is affected by various factors, such as temperature, combustion, and the use of chemicals during treatment processes. Evaluating surface morphology, mass variation, and mechanical, thermal, and chemical properties will play a key role, as MP morphology in sewage sludge is affected by various factors, such as the sludge treatment process [21].

Management of sludge from WWTPs in accordance with environmental principles is an extremely topical issue for sustainable development. This is directly related not only to the construction of WWTPs, but also to the use of sludge as an alternative energy source (energy resource) and/or soil improver (raw material resource) [24]. When used as a soil improver, an extremely important pretreatment process is the decontamination and deworming of sludge from WWTPs. Pathogenic microorganisms and worm eggs can be carried by the sludge. Pathogenic microorganisms such as *Salmonella* sp.*, Listeria* sp., *Escherichia coli*, *Campylobacter* sp., *Clostridium* sp., and *Yersinia* sp. are isolated in sludge. These microorganisms have a high capacity to continuously adapt to changes in the survival environment and can be relatively resistant (especially spore-forming species such as *Clostridium perfringens*) [43]. For this reason, they are characterized by different resistance to environmental stresses, which may result in different distribution and behavior during the treatment and sludge removal process [52].

In order for the sludge to be used as a biological fertilizer, several processing steps must be carried out. It is necessary to reduce the water content of the sludge fraction, which includes dewatering, thickening, and stabilization. All these processes can affect the abundance, size, and morphology of MPs in the sludge. This is due to MP’s affinity to bind to organic matter, and thus their ability to form biofilms [44]. In addition, due to its hydrophobic surface and large specific surface area, sewage sludge can easily adsorb MPs on its surface along with microorganisms. A decomposition study of MPs at a Swedish wastewater treatment plant showed that 66% of smaller MP particles (500 mm) were retained in the sludge fraction [32]. This is particularly problematic for topsoil applications, as the smaller parts can pose a greater ecological threat and thus form biofilms. In sewage sludge, microbial community structure can be modulated by qualitative changes in ecological groups within autotrophic and heterotrophic bacteria depending on the source and availability of MP. In addition, the decomposition of MPs into nanoplastics in sewage sludge can lead to the formation of biofilms, which produce reactive oxygen species (ROS). These cause oxidative stress reactions that have acute inhibitory effects on other microbial communities, including key enzymes, metabolic intermediates, and end products [73].

Although MPs are widely detected in aquatic environments, their occurrence in soil ecosystems remains largely unexplored. Biodegradation of MPs in soil generally occurs through microbial colonization of the MPs surface and depolymerization of MPs into mono- and oligomers through enzymatic hydrolysis [22]. MPs entering the soil environment cause it to accumulate, thereby affecting soil properties, soil processes, and biodiversity. Unlike MPs in aquatic environments, MPs in soil relatively quickly secure sorption sites, forming unique communities in the form of biofilms [7].

**Fig. 3:**
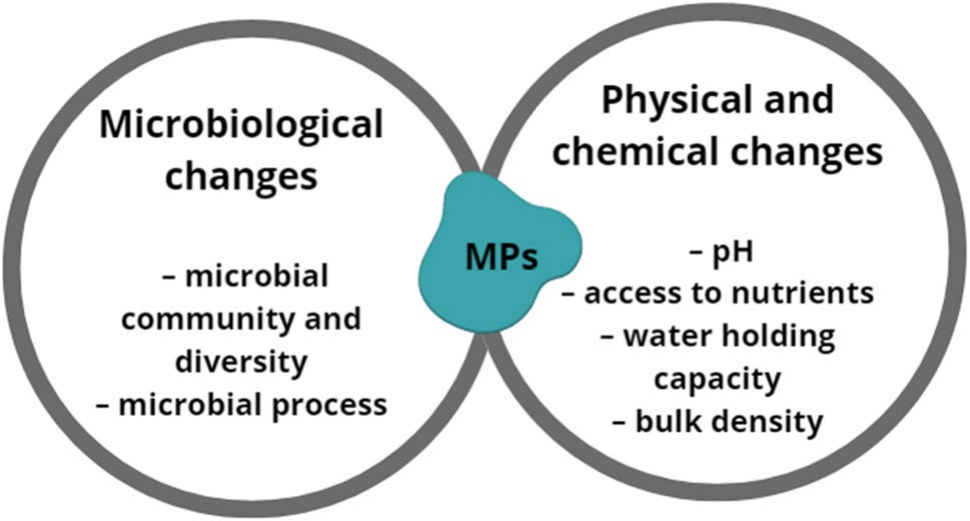
Ecological impact of microplastics on the soil environment

One of the basic soil parameters is the activity of microorganisms. They catalyze many biogeochemical transformations that determine the quality and fertility of the soil and the development of plants, thus ensuring human food security. They can detoxify MPs and break down the material into useful nutrients that support plant growth. However, such effects on plants raise food security concerns in crop production [21]. The impact of microplastic on the soil is schematically shown in Fig. 3. However, such effects on plants raise concerns about food security in crop production. Only a few studies are available on the effects of emerging ecosystem stressors, such as MPs, on soil microorganisms in terrestrial ecosystems. However, recent studies have shown that the physicochemical properties of MPs, such as particle size and polymer density, strongly affect microbial activity in the soil, affecting crop metabolomics (e.g., changes in amino acids, saccharides, and organic acids) and thereby reducing crop biomass [61]. Some studies have shown that no significant effects on soil microbial community structure were observed, while others have observed significant changes in the abundance and diversity of soil microbial communities caused by MPs and the microbes present [71]. Another report showed a reduction in respiration rates and observed significant changes in the rate of root colonization by arbuscular mycorrhizal fungi, suggesting that the presence of MPs can cause changes in microbial functions [49].

Degradation of MPs in soil is a very slow process, mainly due to biodegradation. This is determined by mechanical interaction, oxidation process, and UV radiation. On agricultural soils, plastic MP fragments, which can be the source of sewage sludge, are difficult to decompose and persist for several years. The study presents results showing that the mass loss of MPs in the soil was only 0.1–0.4% after 800 days for PE and 0.4% after 1 year for PP. The main reason that MPs can survive in the soil is poor light and oxygen availability, which ultimately inhibits photo-oxidative degradation [47].

## Conclusion and Future Prospects

The literature review highlights the need for research to deepen understanding and verify the complex interaction between soil ecosystems and MP traits on soil microbial communities. Investigating the formation of biofilms on the surface of plastics and their potential entry into the environment with sewage sludge is important for understanding the impact of these processes on public health and the environment. Literature analysis suggests that biofilms formed on plastic surfaces may play a key role in environmental contamination and pathogen propagation. They consist of a variety of microorganisms that, due to the extracellular polymeric substances, show exceptional resistance to stressors, including disinfectants and antibiotics.

A detailed analysis of the composition of biofilms that form on plastic surfaces is therefore needed to identify all possible microorganisms that can adsorb to the surface of plastics. Subsequently, this will help to better understand the biochemical processes occurring in these structures. Developing research into the mechanisms of biofilm transfer from plastic surfaces to the environment, especially with sewage sludge, can help assess potential risks to public health and aquatic ecosystems. In particular, there should be a focus on developing effective methods to control and prevent biofilm formation on plastic surfaces and their entry into the environment. Regular monitoring of the presence of biofilms on plastic surfaces and their impact on environmental water quality will be key to effective water resource management and public health protection.

The study of biofilm formation on plastic surfaces and its impact on the environment is an important research challenge, the understanding of which can contribute to better pollution management and public health protection. This will allow us to determine how the specific biofilm can affect the biochemical activity and microorganism community in soil that is used for agricultural purposes.

However, there is still a lack of research confirming how applied sewage sludge with MPs can affect specific soil activity processes. The mechanism would need to be identified on a long-term scale. Developing research into the mechanisms of biofilm transfer from plastic surfaces to the environment, especially with sewage sludge, can help assess potential risks to public health and aquatic ecosystems. In particular, there should be a focus on developing effective methods to control and prevent biofilm formation on plastic surfaces and their entry into the environment. Regular monitoring of the presence of biofilms on plastic surfaces and their impact on environmental water quality will be key to effective water resource management and public health protection.

## Data Availability

No datasets were generated or analyzed during the current study.
